# Short-Term Results of the Strengthening and Stretching for Rheumatoid Arthritis of the Hand (SARAH)-Intensive Protocol for Hospitalized Patients With Rheumatoid Arthritis

**DOI:** 10.7759/cureus.116831

**Published:** 2026-09-24

**Authors:** Yuya Takakubo, Miyuki Murakawa, Mizue Kikuchi, Rio Takahashi, Hiromori Sagae, Yoshihiro Wanezaki, Michiaki Takagi

**Affiliations:** 1 Department of Orthopaedics and Rehabilitation, Yamagata University Hospital, Yamagata, JPN; 2 Department of Rehabilitation Medicine, Yamagata University Faculty of Medicine, Yamagata, JPN; 3 Department of Orthopaedic Surgery, Yamagata University Faculty of Medicine, Yamagata, JPN

**Keywords:** in-hospitalized patients, intensive protocol, rheumatoid arthritis (ra), short-term results, strengthening and stretching for rheumatoid arthritis of the hand (sarah)

## Abstract

Background: Advancements in pharmacotherapy have driven a paradigm shift in rheumatoid arthritis (RA) management, subsequently transitioning rehabilitation goals from passive pain control toward proactive functional improvement. This embodies a "rehabilitation treat-to-target (RT2T)" approach. While the Strengthening and Stretching for Rheumatoid Arthritis of the Hand (SARAH) program is an evidence-based self-management regimen, its application in acute inpatient settings remains underexplored. This study aimed to evaluate the short-term effectiveness of an adapted, intensive inpatient version of this program (SARAH-Intensive Protocol (SARAH-IP)).

Methods: This study evaluated 21 hospitalized patients with RA (mean age 69.0 years) prospectively who underwent the SARAH-IP during admission, primarily following orthopedic surgery or systemic medication initiation, at the Department of Orthopaedic Surgery & Rehabilitation, Yamagata University Faculty of Medicine, Japan. The SARAH-IP involved therapist-guided functional training five times weekly, strategically supplemented by patient-led weekend self-exercises and continuous structured feedback. Primary outcomes, including grip strength (dominant and non-dominant) and Quick Disabilities of the Arm, Shoulder, and Hand (Q-DASH) scores, were assessed at baseline and discharge.

Results: The SARAH-IP group achieved an overall 20/21 (95%) improvement rate in grip strength and Q-DASH scores, translating directly to enhanced activities of daily living (ADL). Crucially, the SARAH-IP cohort exhibited significant bilateral enhancements.

Conclusions: The SARAH-IP is an effective short-term inpatient rehabilitation strategy for RA, conferring comprehensive functional benefits to both dominant and non-dominant upper extremities. By integrating continuous therapist feedback with self-managed exercises, this intensive protocol effectively bridges inpatient intervention with post-discharge self-care. Future research should evaluate the long-term adherence and efficacy of this regimen in broader patient populations.

## Introduction

The treatment of rheumatoid arthritis (RA) has undergone a paradigm shift, primarily driven by advancements in pharmacotherapy. The therapeutic goals have transitioned from mere pain control to the prevention of bone and joint destruction and the preservation of physical function [1,2,3]. Consequently, rehabilitation therapy for RA is now expected to facilitate the achievement of higher levels of activities of daily living (ADL) and quality of life (QOL).

Rehabilitation for RA primarily comprises physical therapy and occupational therapy (OT). Standard OT for RA typically encompasses muscle strengthening, applied motor training, orthosis fabrication, and joint protection education [3]. In 2015, Lamb et al. developed the Strengthening and Stretching for Rheumatoid Arthritis of the Hand (SARAH) program, a 12-week self-management regimen for home-based hand exercises [4]. The SARAH protocol consists of 11 straightforward hand exercises: seven mobility exercises and four strengthening exercises. Patients are instructed to maintain a daily checklist, document facilitators and barriers to program execution prior to their sixth outpatient session, and discuss evaluations and potential solutions with their therapist. A defining characteristic of the SARAH program is that therapists do not merely prescribe exercises; rather, patients and therapists collaboratively establish daily life goals, identify facilitators for sustained participation, and formulate solutions to overcome barriers.

This methodology embodies a "rehabilitation treat-to-target (RT2T)" approach [5]. The SARAH program has proven to be both clinically effective and cost-efficient, earning a recommendation from the UK National Institute for Health and Care Excellence (NICE) guidelines for adult RA management [4]. Despite these therapeutic advances, RA frequently leads to functional decline, particularly in the hands and upper limbs [4,5]. Therefore, effective rehabilitation remains crucial for managing symptoms and preserving function in patients with RA.

In the present study, we implemented an intensive adaptation of this program, termed the SARAH-Intensive Protocol (SARAH-IP), for hospitalized patients with RA prospectively. The primary objective of this study was to evaluate the short-term effectiveness of the SARAH-IP in a hospitalized RA cohort.

## Materials and methods

Participants

This was a prospective single-arm intervention study in a single institute and included 21 hospitalized patients diagnosed with RA according to the 2010 ACR/EULAR classification criteria and the 1987 American College of Rheumatology criteria, who were prescribed OT during their hospitalization [1,2]. Between May 2021 and April 2022, the inaugural year of the SARAH-IP study, 55 patients with RA were admitted to the Department of Orthopaedic Surgery & Rehabilitation, Yamagata University Faculty of Medicine, Japan, for surgery or the initiation of biological therapies. From this cohort, we included 21 patients who provided informed consent to participate and were deemed medically eligible for the SARAH-IP by their attending physicians. The primary reasons for hospitalization among the included cohort were lower extremity orthopedic surgeries for 20 patients (specifically, 11 total knee arthroplasties, three total hip arthroplasties, and six forefoot joint arthroplasties) and the initiation of systemic medication (abatacept induction) for one patient. Exclusion criteria were defined as follows: patients without a confirmed diagnosis of RA, those undergoing surgical procedures other than lower limb arthroplasty, patients who declined participation in the SARAH-IP, and those considered clinically ineligible by their attending physicians (Figure 1).

**Figure 1:**
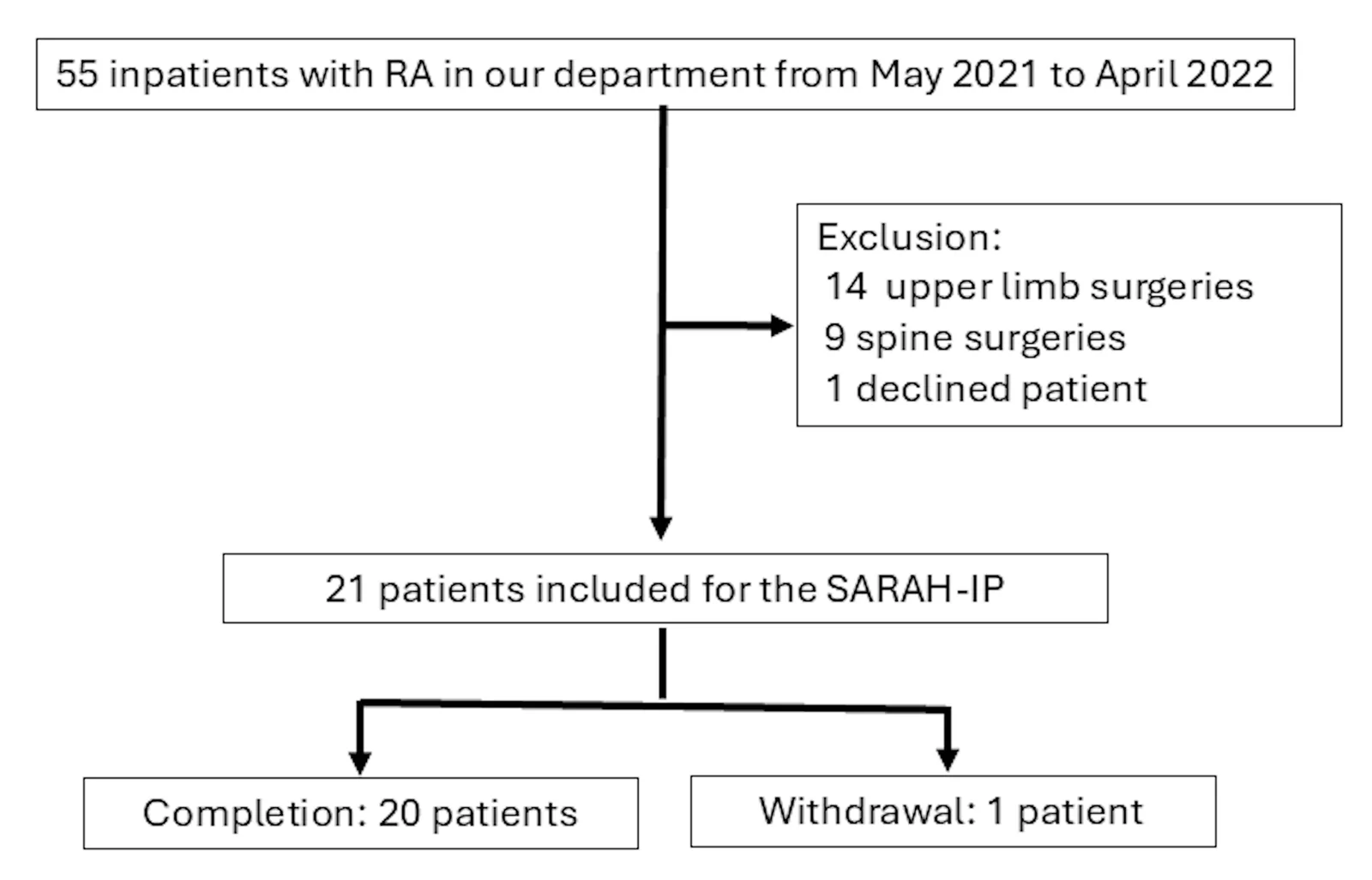
CONSORT diagram The CONSORT diagram shows the study design, comprising  inpatients with RA in our department who were registered in the study population

Based on these criteria, a total of 34 patients were excluded. Utilizing G*Power 3.1 (Universitat Kiel, Germany) and setting the effect size to 0.6, alpha error to 0.05, and power to 95%, the required total sample size was determined to be 20 participants [6]. Therefore, the study aimed to recruit at least 20 participants. The implementation of the SARAH-IP was approved by the Institutional Ethics Committee of Yamagata University (approval no. 2021-S-105).

SARAH-Intensive Protocol (SARAH-IP)

The original SARAH program, which forms the foundation of our modified protocol, comprises seven distinct mobility exercises designed to improve range of motion (ROM) and four specific exercises aimed at enhancing muscle strength (Figure 2) [4,5].

**Figure 2:**
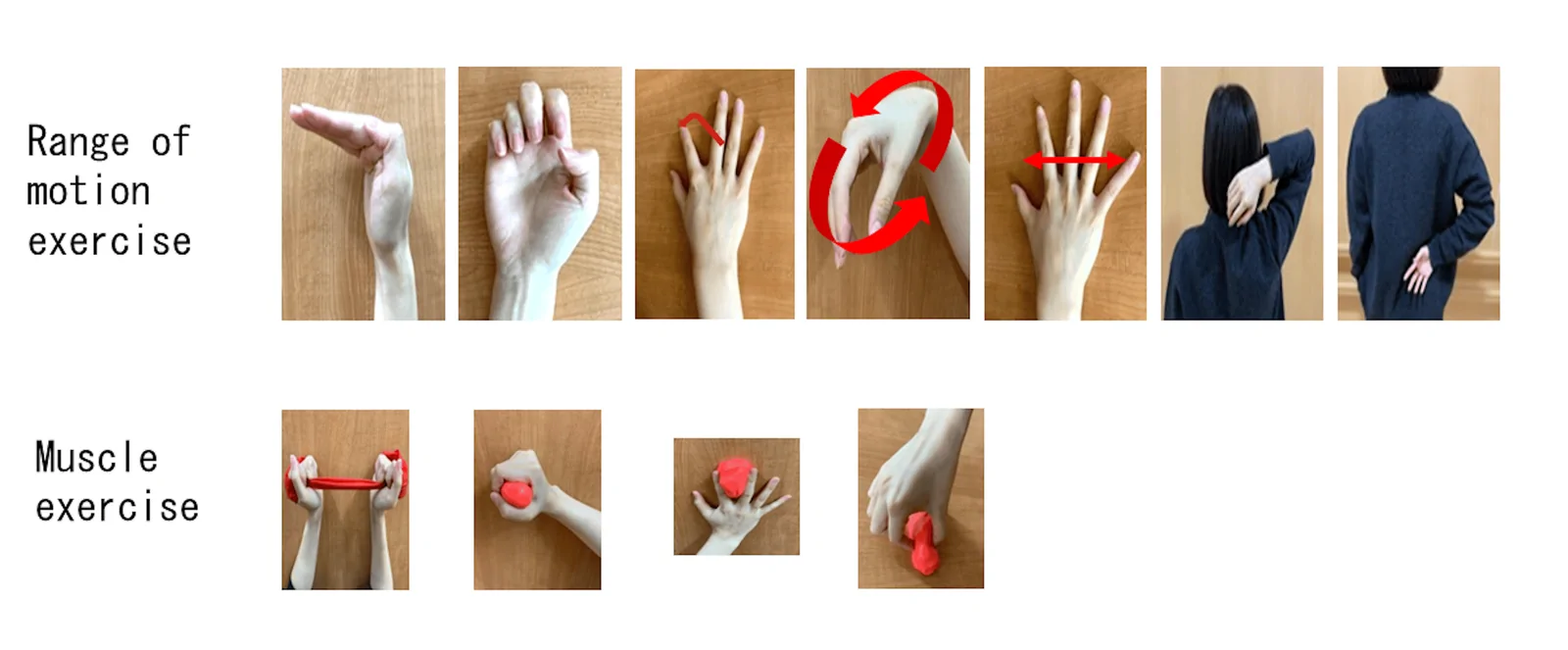
Strengthening and Stretching for Rheumatoid Arthritis of the Hand (SARAH) program The SARAH program consists of seven mobility exercises and four strengthening exercises. While the original SARAH regimen typically involved a 12-week period of home-based self-exercises supplemented by only six outpatient occupational therapy (OT) follow-up sessions, the SARAH-IP implemented at our facility involved dedicated exercise instruction and practical sessions conducted five times per week during the patient's inpatient OT.

While the original regimen typically involved a 12-week period of home-based self-exercises supplemented by only six outpatient follow-up sessions, the SARAH-IP implemented at our facility involved dedicated exercise instruction and practical sessions conducted five times per week during the patient's inpatient OT, commencing after surgery or the initiation of medication. A comprehensive assessment of each patient's baseline exercise capacity was conducted prior to implementation. Patients were actively encouraged to perform the SARAH-IP independently in their hospital rooms over the weekends when formal sessions were not scheduled. Crucially, at the beginning of each week, therapists provided detailed feedback regarding the patients' weekend self-exercise performance, utilizing the SARAH patient guide self-diary to reinforce proper technique and sustain motivation.

The whole OT session lasted 40 minutes and encompassed functional training, ADL training, and RA-specific patient education. Functional training consisted of ROM exercises (five to 10 repetitions per joint) tailored to the patient's joint status, alongside isometric muscle strengthening exercises (held for five seconds, 10-20 repetitions). ADL training focused on joint protection techniques, energy conservation strategies, the utilization of self-help and assistive devices, and necessary lifestyle modifications. Educational pamphlets were distributed to inform patients about RA pathology, the characteristics of joint deformities, and pain management strategies. The self-management component required patients to independently continue the functional exercises learned during sessions and to maintain a lifestyle adhering to joint protection principles.

At the initial intervention and at discharge, two primary outcomes were assessed: scores from the Quick Disabilities of the Arm, Shoulder and Hand (Q-DASH) questionnaire, and grip strength. In a resting, seated position, grip strength was measured twice for both the dominant and non-dominant hands using a 300 mmHg mercury sphygmomanometer, and the mean value of the two measurements was recorded as the final grip strength [7].

Statistical analysis

Statistical comparisons were performed using the Wilcoxon signed-rank test via IBM SPSS Statistics 25 software (SPSS Institute Inc., Chicago, IL, USA). A p-value of <0.05 was considered statistically significant.

## Results

The SARAH-IP cohort comprised 17 females and four males, with a mean age of 69.0 ± 6.3 years. The mean disease duration of RA was 17.4 ± 12.3 years, and the mean length of hospitalization was 25.3 ± 11.5 days. The participants received an average of 13.8 ± 5.7 OT intervention sessions during their stay, equating to a rate of 0.6 ± 0.1 sessions per day. Disease activity, as assessed by the DAS28-ESR(4) (mean score: 3.0 ± 0.9), indicated low disease activity in 10 patients and moderate disease activity in 11 patients. Concomitant medications included biologics (eight patients: four abatacept, three etanercept, and one adalimumab), methotrexate (MTX; 10 patients, mean dose: 8.6 mg/day), sulfasalazine (SASP; three patients, mean dose: 1000 mg/day), and prednisolone (PSL; four patients, mean dose: 2.1 mg/day) (Table 1).

**Table 1:** Characteristics of Strengthening and Stretching for Rheumatoid Arthritis of the Hand (SARAH) intensive protocol (IP) group

		SARAH IP group
Case		21
Ages	(old-year)	69.0±6.3
Sex	Male:female	4:17
Duration of rheumatoid arthritis	(months)	17.4±12.3
Surgery		TKA 11, THA 3, Forefoot 6
DAS28ESR4		3.0±0.9
	High	0
	Moderate	11
	Low	10
Steinbrockers	Stage	Ⅲ; 4, Ⅳ; 17
	Class	2; 4, 3; 17
Medication		
Biologics	(patients)	8/21 (38%)
Methotrexate (MTX)	(patients)	10/21 (48%)
Mean dosage of MTX	(mg/week)	8.6±2.3
Salazosulfapyridine (SASP)	(patients)	3/21 (14 %)
Mean dosage of SASP	(mg/day)	1000±0
Prednisolone (PSL)	(patients)	4/21 (19%)
Mean dosage of PSL	(mg/day)	2.1±0.8
Intervention times by a therapist	(times/intervention)	13.8±5.7
Duration of intervention	(days)	25.3±11.5
Intervention average	(times/day)	0.6±0.1

Significant improvements in motor function were observed at discharge compared to baseline status in the SARAH-IP group. The mean grip strength of the dominant hand improved from 140.6 ± 43.1 at the initial intervention to 150.8 ± 48.1* at discharge (p < 0.05, statistically significant). The mean grip strength of the non-dominant hand improved from 128.0 ± 42.9 at the initial intervention to 145.4 ± 46.1* at discharge (p < 0.05, statistically significant). The mean Q-DASH scores improved from 28.4 ± 18.5 at the initial intervention to 23.8 ± 12.9 at discharge (p < 0.1, not statistically significant). Changes in the mean grip strength of the dominant and non-dominant hands and Q-DASH scores are summarized in Table 2.

**Table 2:** Changes in grip power and Quick Disabilities of the Arm, Shoulder, and Hand (Q-DASH) scores from the initial intervention to discharge in Strengthening and Stretching for Rheumatoid Arthritis of the Hand (SARAH) Intensive Protocol (IP) group Statistical comparisons were performed using the Wilcoxon signed-rank test via IBM SPSS Statistics 25 software (SPSS Institute Inc., Chicago, IL, USA). *A p-value of <0.05 was considered statistically significant.

SARAH IP group	At the initial intervention	At discharge	Value of change
Grip power (mmHg), dominant hand	140.6 ± 43.1	150.8 ± 48.1*	10.1 ± 10.7
Grip power (mmHg), non-dominant hand	128.0 ± 42.9	145.4 ± 46.1*	17.4 ± 22.5
Q–DASH (score)	28.4 ± 18.5	23.8 ± 12.9	-4.5 ± 8.6

Overall, improvements in grip strength and Q-DASH scores were achieved in 20 of the 21 patients in the SARAH-IP group, representing a 95% improvement rate. Notable specific functional gains in ADL were documented: three patients remarkably regained the ability to independently open and close plastic bottles, two patients demonstrated enhanced squeezing capabilities, and one patient experienced improved dexterity in using a knife. In addition, the SARAH-IP group demonstrated improvements in grip strength for both the dominant and non-dominant hands.

However, one male patient in his 70s, who had a complex medical history including depression and reported poor motivation and comprehension, did not exhibit functional improvement following the SARAH-IP intervention, and the intervention was subsequently discontinued at his request.

## Discussion

Our study demonstrates the short-term efficacy of the SARAH-IP in improving hand function among hospitalized patients with RA prospectively. These findings corroborate previous reports indicating that SARAH exercises, when guided by a therapist and practiced consistently, facilitate the acquisition of proper exercise techniques, subsequently leading to improvements in physical function and upper limb utility [4,5,8,9].

The SARAH-IP protocol prescribed more detailed ROM and muscle strengthening exercises, specifically incorporating a greater number of targeted interventions for the MCP and PIP joints. Previous reports indicate that grip strength is generally weaker in patients with RA compared to healthy individuals, and this tendency is even more pronounced on the non-dominant side [10]. However, because similar improvements were observed in the dominant and non-dominant hands, we hypothesize that the comprehensive and meticulously detailed nature of the SARAH-IP program primarily facilitated the unique improvements observed in the non-dominant hands.

The treatment of RA has undergone a paradigm shift, driven largely by advances in pharmacotherapy, such as the introduction of biologics and Janus kinase (JAK) inhibitors [2]. The primary therapeutic objective has transitioned from mere pain palliation to the prevention of osteoarticular destruction and the preservation of physical function. Consequently, the aims of RA rehabilitation have concurrently shifted from conventional pain relief and deformity prevention to proactive joint protection until remission is achieved, followed by functional optimization once remission is sustained [2]. Despite the widespread utilization of cutting-edge pharmacotherapies, 10-20% of patients still experience inadequate therapeutic responses or difficulties in maintaining treatment, and a subset continues to exhibit progressive osteoarticular damage [2]. Furthermore, reflecting the realities of a super-aging society, the RA population is aging, and the incidence of elderly-onset RA is increasing. These elderly patients frequently present with diverse comorbidities-including hypertension, hyperlipidemia, cardiovascular disease, pulmonary disease, diabetes mellitus, chronic kidney disease, malignancies, osteoporosis, and osteoarthritis-alongside physical impairments, frailty, locomotive syndrome, sarcopenia, dementia, and compromised immune function. These factors, coupled with concerns regarding polypharmacy, often restrict pharmacological options, forcing clinicians to lower the therapeutic target from clinical remission to low disease activity. For patients facing such pharmacological limitations, rehabilitation plays a profoundly critical role in preserving function and alleviating rheumatic symptoms.

Both the EULAR recommendations and the Japanese clinical practice guidelines for RA emphasize the utility of exercise therapy within RA rehabilitation to enhance ADL and QOL [11,12]. Nevertheless, studies indicate that RA patients still engage in fewer daily steps and less weekly exercise compared to the general population [13]. Concurrently, the landscape of RA rehabilitation in Japan is undergoing significant changes. According to the Rheumatism White Paper, the proportion of patients receiving formal rehabilitation decreased from 37% in 2005 to 23.6% in 2020 [12,13]. This decline can be attributed to reduced demand and altered indications for rehabilitation among both patients and healthcare providers-a direct consequence of the pharmacological paradigm shift-as well as a reduction in outpatient rehabilitation facilities following revisions to the national medical remuneration system. Under these circumstances, the SARAH exercise program, which fundamentally prioritizes patient self-management and the targeted improvement of upper limb function, holds tremendous clinical potential [4,5].

The paradigm shift in RA treatment necessitates innovative approaches to rheumatic rehabilitation in the biologics era. By operationalizing the concept of "Rehabilitation Treat-to-Target (RT2T)," we aim to maximize functional recovery and prevent the recurrence of deformities, ultimately enhancing long-term outcomes and patient satisfaction [14].

Notably, the original SARAH trial reported significant improvements in hand muscle strength, including grip force, within the exercise cohort [3]. Among those who completed the extended follow-up, the mean change in overall hand function at 12 months remained superior in the exercise group compared to usual care. However, this significant difference did not persist at the final extended follow-up beyond two years [8,9]. A major distinction is that the original SARAH program involved only six outpatient OT sessions, whereas the SARAH-IP delivered an average of 13.8 sessions, representing more than double the intervention frequency. The short-term improvements in grip strength (significant) and Q-DASH scores (non-significant) observed in our cohort underscore the utility of this adapted program. The observation that 88% of our hospitalized RA patients demonstrated short-term benefits from the SARAH-IP is compelling, highlighting its value as an inpatient rehabilitation strategy. Furthermore, the tangible functional gains directly translated to enhanced independence and QOL for these individuals. The structural design of the SARAH-IP -particularly the incorporation of weekend self-exercises appears to effectively bridge the gap between inpatient rehabilitation and self-managed post-discharge care. This frequent, short-term, intensive approach is crucial for solidifying the therapeutic benefits achieved during hospitalization.

Despite these encouraging short-term results, a critical future challenge lies in developing robust strategies to maximize long-term exercise adherence [4,5]. The long-term follow-up of the original SARAH trial indicated that while immediate benefits were evident, the positive effects on hand function diminished after 12 months, directly coinciding with reduced self-reported exercise adherence. Recently, an online iteration of the program (mySARAH) has demonstrated not only promising efficacy comparable to the original trial but also superior patient retention rates [8,9].

Future research must explore adaptive strategies and supportive interventions tailored to these specific patient needs. Rigorous evaluations of the long-term efficacy and adherence rates of the SARAH-IP in larger, more diverse patient populations are essential. A hybrid model integrating face-to-face instruction with digital or online versions of the SARAH program may offer a viable solution for broader accessibility and sustained patient engagement.

This study is subject to several limitations. First, the sample size was relatively small. Second, the follow-up period was limited to the short term. Third, this was a prospective single-arm intervention study in a single institute. Future multicenter, prospective, collaborative studies are warranted to further validate these findings.

## Conclusions

The paradigm shift in RA treatment necessitates innovative approaches to rheumatic rehabilitation in the biologics era. Our findings demonstrate that the SARAH-IP is a highly effective short-term inpatient rehabilitation strategy, conferring comprehensive functional benefits to both the dominant and non-dominant upper extremities. By integrating continuous therapist feedback with self-managed exercises, this intensive protocol effectively bridges inpatient intervention with post-discharge self-care. Although this pilot study is limited by a small sample size and a short-term period, a critical future challenge lies in developing robust strategies to maximize long-term exercise adherence.

## References

[1] Aletaha D, Neogi T, Silman AJ (2010). 2010 rheumatoid arthritis classification criteria: an American College of Rheumatology/European League Against Rheumatism collaborative initiative. Ann Rheum Dis.

[2] Smolen JS, Landewé RB, Bergstra SA (2023). EULAR recommendations for the management of rheumatoid arthritis with synthetic and biological disease-modifying antirheumatic drugs: 2022 update. Ann Rheum Dis.

[3] Harigai M, Kaneko Y, Tanaka E (2025). 2024 update of the Japan College of Rheumatology Clinical Practice Guidelines for the management of rheumatoid arthritis: secondary publication. Mod Rheumatol.

[4] Lamb SE, Williamson EM, Heine PJ (2015). Exercises to improve function of the rheumatoid hand (SARAH): a randomised controlled trial. Lancet.

[5] Takakubo Y, Murakawa M, Yang S (2024). Application of SARAH-modified exercise program for a late patient with rheumatoid arthritis, a case report [Article in Japanese]. Kansetsu no Geka.

[6] Mascha EJ, Vetter TR (2018). Significance, errors, power, and sample size: the blocking and tackling of statistics. Anesth Analg.

[7] Moradi A, Menendez ME, Kachooei AR, Isakov A, Ring D (2016). Update of the Quick DASH Questionnaire to account for modern technology. Hand (N Y).

[8] Williamson E, McConkey C, Heine P, Dosanjh S, Williams M, Lamb SE (2017). Hand exercises for patients with rheumatoid arthritis: an extended follow-up of the SARAH randomised controlled trial. BMJ Open.

[9] Srikesavan C, Williamson E, Lamb SE (2025). The online version of an evidence-based hand exercise programme for people with rheumatoid arthritis: An effectiveness-implementation study. J Hand Ther.

[10] Fraser A, Vallow J, Preston A, Cooper RG (1999). Predicting 'normal' grip strength for rheumatoid arthritis patients. Rheumatology (Oxford).

[11] Nagy G, Roodenrijs NM, Welsing PM (2022). EULAR points to consider for the management of difficult-to-treat rheumatoid arthritis. Ann Rheum Dis.

[12] Gwinnutt JM, Wieczorek M, Balanescu A (2023). 2021 EULAR recommendations regarding lifestyle behaviours and work participation to prevent progression of rheumatic and musculoskeletal diseases. Ann Rheum Dis.

[13] Okano T, Inui K, Tada M (2017). Loss of lean body mass affects low bone mineral density in patients with rheumatoid arthritis - results from the TOMORROW study. Mod Rheumatol.

[14] Takakubo Y, Wanezaki T, Takagi M (2020). Lower extremity surgery and rehabilitation therapy for rheumatoid arthritis [Article in Japanese]. Jpn J Rehabil Med.

